# Turner syndrome with pulmonary arteriovenous malformation: a case report

**DOI:** 10.3389/fcvm.2025.1603250

**Published:** 2025-08-28

**Authors:** Huibin Guo, Hongqiao Chen, Sihao Chen, Shilong Tang

**Affiliations:** ^1^ Department of Radiology, University-Town Hospital of Chongqing Medical University, Chongqing, China; ^2^ Department of Radiology, Children's Hospital of Chongqing Medical University, National Clinical Research Center for Child Health and Disorders, Ministry of Education Key Laboratory of Child Development and Disorders, Chongqing Key Laboratory of Pediatrics, Chongqing, China

**Keywords:** TS, PAVM, children, CTA, heart

## Abstract

Turner syndrome (TS) is the most common sex chromosome abnormality disorder, caused by complete or partial absence of the X chromosome, its clinical manifestations primarily include short stature, gonadal dysgenesis, and characteristic cardiovascular malformations, pediatric cardiologists pay particular attention to coarctation of the aorta (CoA), which occurs in 15%–30% of TS patients and represents a life-threatening condition requiring prioritized screening during the neonatal and childhood periods (1– 3). Furthermore, due to lymphatic system developmental abnormalities, TS patients also face elevated risks of aortic root dilation, bicuspid aortic valve, and vascular structural anomalies (3). Pulmonary arteriovenous malformation (PAVM) is a rare pulmonary vascular anomaly, with an estimated prevalence of approximately 1 in 50,000 in the general population (1–3). Although the exact prevalence of PAVM in TS patients remains unclear, case series suggest a significantly elevated risk compared to the general population (estimated risk ratio: 5- to 10-fold), this association may be attributed to defective vascular elastic fiber development and dysregulated angiogenic signaling pathways in TS patients (4, 5). Here, we report the first documented case of TS complicated by PAVM, aiming to enhance clinicians' awareness of this rare comorbidity and provide evidence-based diagnostic and therapeutic recommendations.

## Introduction

Turner syndrome (TS) is the most common sex chromosome abnormality disorder, caused by complete or partial absence of the X chromosome, its clinical manifestations primarily include short stature, gonadal dysgenesis, and characteristic cardiovascular malformations, pediatric cardiologists pay particular attention to coarctation of the aorta (CoA), which occurs in 15%–30% of TS patients and represents a life-threatening condition requiring prioritized screening during the neonatal and childhood periods (1–3). Furthermore, due to lymphatic system developmental abnormalities, TS patients also face elevated risks of aortic root dilation, bicuspid aortic valve, and vascular structural anomalies (3).

Pulmonary arteriovenous malformation (PAVM) is a rare pulmonary vascular anomaly, with an estimated prevalence of approximately 1 in 50,000 in the general population, PAVM can reduce blood oxygen saturation, increase cardiac output, lead to embolism or hemorrhage, and in the long term, may result in hypoxemia and secondary polycythemia (1–3). Although the exact prevalence of PAVM in TS patients remains unclear, case series suggest a significantly elevated risk compared to the general population (estimated risk ratio: 5- to 10-fold), this association may be attributed to defective vascular elastic fiber development and dysregulated angiogenic signaling pathways in TS patients (4, 5).

Due to the unique circulatory and lymphatic system abnormalities in patients with Turner syndrome,they have an increased risk of arteriovenous malformations, which may manifest as more severe or more subtle clinical symptoms. Here we present a rare case of Turner Syndrome with pulmonary arteriovenous malformation, to date, no previous cases of this kind have been published, we share this case to raise clinical awareness of this rare variation and emphasize the importance of early diagnosis.

## Case report

**Figure 1:**
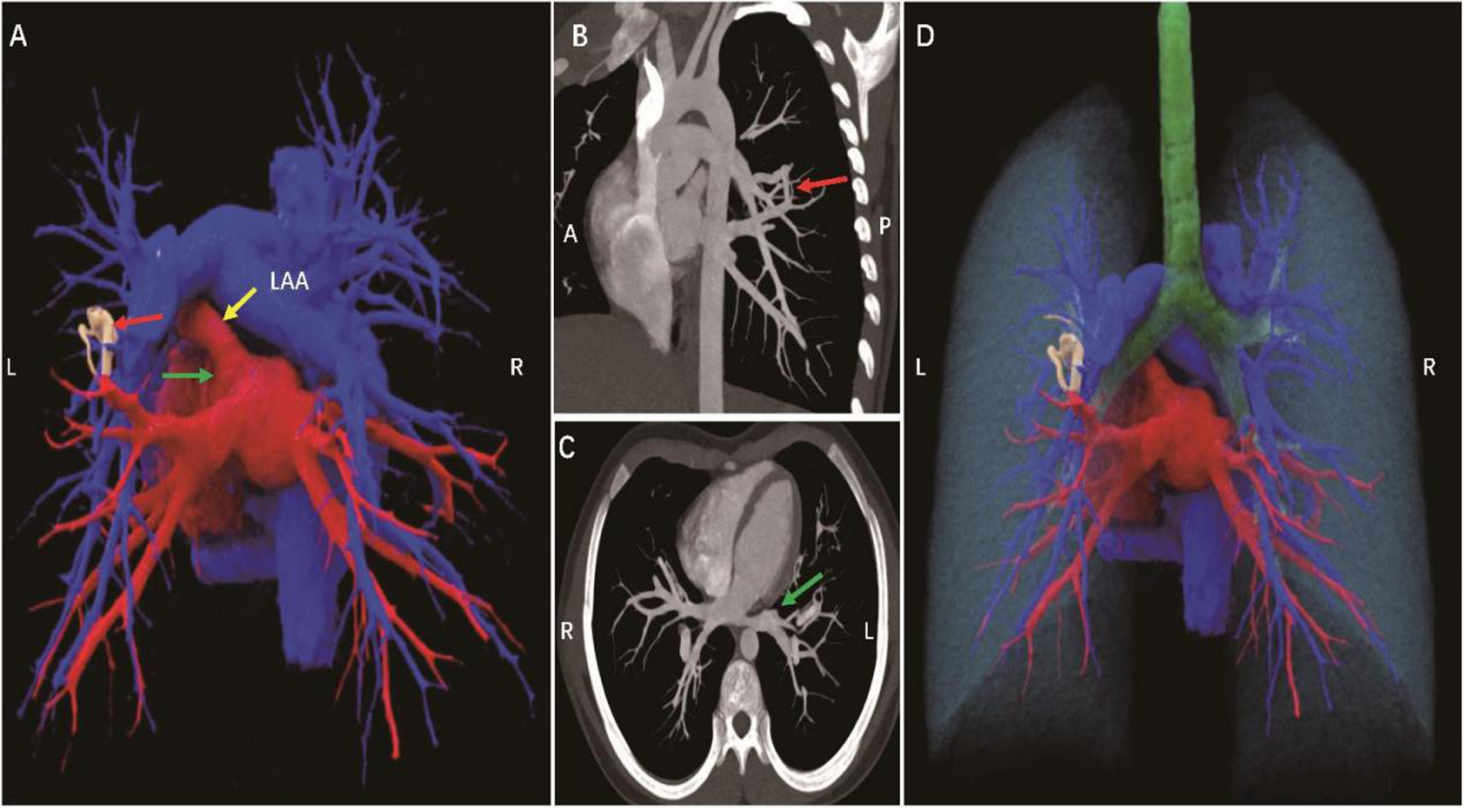
A 13-year-old female patient, pulmonary arteriovenous malformation **(A)**. Cinematic rendering shows an abnormal vascular shadow with tortuous running and uneven thickness between the left lower pulmonary artery and the proximal left upper pulmonary vein (red arrow) **(B,C)**. CT MIP images show that the pulmonary artery is directly connected to the vein, forming a traffic (red arrow), and the left upper pulmonary vein is not seen (green arrow). **(D)** Cinematic rendering clearly shows the spatial position relationship between the lung, pulmonary artery, pulmonary vein, trachea and the lesion site. A, anterior; P, posterior; L, left; R, right; LAA, left atrial appendage.

A 13-year-old female patient with Turner syndrome (TS), undergoing growth hormone and estrogen replacement therapy, was found to have a grade 3/6 systolic murmur at the left sternal border (second to third intercostal space) during routine physical examination. She had no prior history of cyanosis or exercise intolerance, with resting oxygen saturation (SpO_2_) of 96% (declining to 94% post-exertion). Transthoracic echocardiography revealed anomalous drainage of the left upper pulmonary vein, and computed tomography angiography (CTA) confirmed the presence of a left lower lobe pulmonary arteriovenous malformation (PAVM) with a feeding artery diameter of 5 mm (Figure 1, Supplementary Movie). According to current PAVM management guidelines (6), lesions <3 mm in diameter without symptoms may be managed conservatively with surveillance. Thus, a follow-up protocol was established, including CTA and transthoracic echocardiography (TTE) every 6 months. Notably, the patient showed no evidence of coarctation of the aorta or other aortic pathologies (normal aortic arch and root dimensions), ruling out the most common cardiovascular malformations associated with TS. The timing of murmur onset during puberty, coupled with CTA findings demonstrating congenital vascular malformation characteristics, suggests that this PAVM likely represents a congenital lesion that became hemodynamically significant due to adolescent circulatory changes.

## Discussion

This case highlights the need for clinicians to maintain vigilance for atypical vascular malformations when evaluating Turner syndrome (TS) patients, particularly in the presence of: (1) unexplained cardiac murmurs; (2) abnormal oxygen saturation; or (3) decreased exercise tolerance. Computed tomography angiography (CTA) represents the diagnostic gold standard for pulmonary arteriovenous malformations (PAVMs), while management decisions should integrate lesion size, symptomatology, and complication risks.

PAVMs carry significant mortality if untreated. Endovascular embolization serves as first-line therapy for all PAVMs with feeding vessels >3 mm in diameter. Current guidelines recommend proactive intervention for all technically amenable PAVMs, particularly symptomatic cases or those demonstrating right-to-left shunting, regardless of feeding artery diameter (7). Asymptomatic small lesions (such as in this case) warrant regular imaging surveillance to monitor progression.

Notably, the literature contains extremely few reports of TS coexisting with PAVM. This rarity underscores both the exceptional nature of our case and suggests potential underrecognition of pulmonary vascular anomalies in TS patients, whose vascular screening protocols may currently focus predominantly on aortic pathologies. Future large-scale cohort studies are needed to establish the true prevalence and natural history of PAVMs in TS populations.

This study has several limitations: As a single case report, it cannot establish the true association strength between TS and PAVM;the absence of long-term follow-up data precludes accurate assessment of lesion progression risk; genetic sequencing was not performed, leaving potential synergistic genetic mutations contributing to vascular maldevelopment undetermined. Future studies should accumulate additional cases and incorporate genetic analyses to elucidate the molecular mechanisms underlying rare vascular anomalies in TS patients.

## Conclusion

This case represents the first confirmed report of pulmonary arteriovenous malformation (PAVM) co-occurring with Turner syndrome (TS), suggesting that PAVM should be included in the differential diagnosis of cardiovascular complications when evaluating TS patients. For patients presenting with cardiac murmurs or hypoxemia, contrast-enhanced computed tomography angiography (CTA) is recommended to rule out PAVM. In the absence of TS-specific evidence, therapeutic decisions should be based on existing PAVM management guidelines for the general population, while incorporating individualized treatment strategies tailored to each patient's clinical profile.

## Data Availability

The original contributions presented in the study are included in the article/Supplementary Material, further inquiries can be directed to the corresponding author.
